# Supplementary figures and images for: Chemical Diversity between Three Graminoid Plants Found in Western Kenya Analyzed by Headspace Solid-Phase Microextraction Gas Chromatography–Mass Spectrometry (HS-SPME-GC-MS)
Source: Plants (Basel). 2021 Nov 10;10(11):2423. doi: 10.3390/plants10112423 (PMC8617917; doi:10.3390/plants10112423)

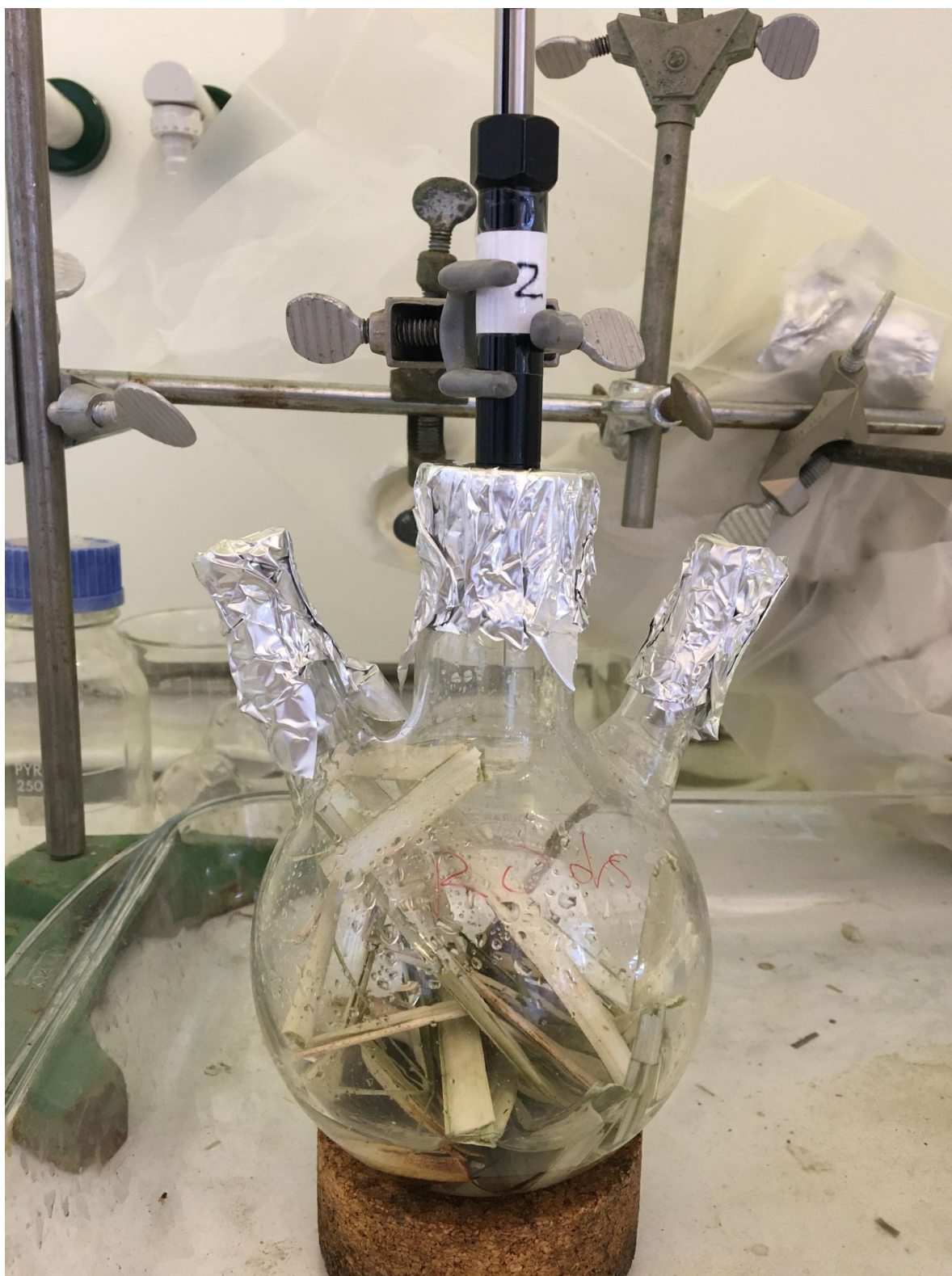

Supplement: Supplementary file 1 [file plants-10-02423-s001.zip › Figure S1.pdf]
